# Supplementary material for: An investigation of how perceived smart tourism technologies affect tourists’ well-being in marine tourism
Source: PLoS One. 2023 Aug 25;18(8):e0290539. doi: 10.1371/journal.pone.0290539 (PMC10456169; doi:10.1371/journal.pone.0290539)
Supplement: Attachment — Submitted filename: PONE-Review-0618.docx [file pone.0290539.s002.docx]

This research discusses how perceived smart tourism technologies affect tourists' well-being. However, some contents in the manuscript did not present logically, especially reviewer can hardly see the research gap and implications through the manuscript. Additionally, the method of data collection was unclear and some paragraphs are problematics. Therefore, the following comments are provided and hope it helps improve the quality of this manuscript.

**Introduction**

1. P3, “Although hedonic well-being with … personal potential, virtues, character strengths, self-growth, self-fulfillment”. The authors only describe which aspects it pays more attention to in the few tourism literatures that study eudaimonic well-being, but do not discuss the gaps between this study and existing research. Please see some of the examples below:

- Li, T. E., & Chan, E. T. H. (2017). Diaspora tourism and well-being: A eudaimonic view. *Annals of Tourism Research*, *63*, 205-206.
- Hao, F., & Xiao, H. (2021). Residential tourism and eudaimonic well-being: A ‘value-adding’analysis. *Annals of Tourism Research*, *87*, 103150.
- Su, L., Tang, B., & Nawijn, J. (2020). Eudaimonic and hedonic well-being pattern changes: Intensity and activity. *Annals of Tourism Research*, *84*, 103008.
- Huang, X., Wang, P., & Wu, L. (2023). Well-being Through Transformation: An Integrative Framework of Transformative Tourism Experiences and Hedonic Versus Eudaimonic Well-being. *Journal of Travel Research*, 00472875231171670.
- Rahmani, K., Gnoth, J., & Mather, D. (2018). Hedonic and eudaimonic well-being: A psycholinguistic view. *Tourism Management*, *69*, 155-166.
- Chang, L., Moyle, B. D., Dupre, K., Filep, S., & Vada, S. (2022). Progress in Research on Seniors' well-being in tourism: A systematic review. *Tourism Management Perspectives*, *44*, 101040.

**Literature review**

2. P9-P10, “The integration of smart tourism technologies…. In light of this, the following hypotheses were developed”. The discussion on the relationship between perceived smart tourism technology and tourist well-being in this paragraph is relatively weak. The examples given by the authors do not well support the hypothesis.

**Research method**

3. P13-P14, “This paper draws on previously validated scales…are based on research conducted by Diener”. Reviewers cannot see the scale for this study. It is recommended to show the complete scale.

**Data collection**

4. P14, “This study is aimed at people…Table 1 lists more details of the sample for this study”. Why did the authors use a combination of online and offline methods to collect data? After collecting these two parts of the data, was there a t-test performed? How are offline questionnaires collected?
5. P14, Table1: It is inappropriate to collect questionnaires from participants younger than 18 years of age.

**Data analysis and results**

6. Table 2, Table 4 and Table 5: The authors did not explain what each abbreviation means.

**Theoretical contributions**

7. P21, “Firstly, while most of the existing research…on tourist satisfaction and willingness to revisit”. This sentence lacks citations and is not convincing enough.
